# Assessment of the Lipophilicity of Indole Derivatives of Betulin and Their Toxicity in a Zebrafish Model

**DOI:** 10.3390/molecules29184408

**Published:** 2024-09-16

**Authors:** Zuzanna Rzepka, Katarzyna Bober-Majnusz, Justyna Magdalena Hermanowicz, Ewa Bębenek, Elwira Chrobak, Arkadiusz Surażyński, Dorota Wrześniok

**Affiliations:** 1 Department of Pharmaceutical Chemistry, Faculty of Pharmaceutical Sciences in Sosnowiec, Medical University of Silesia in Katowice, 4 Jagiellońska, 41-200 Sosnowiec, Poland; zrzepka@sum.edu.pl; 2 Department of Analytical Chemistry, Faculty of Pharmaceutical Sciences in Sosnowiec, Medical University of Silesia in Katowice, 41-200 Sosnowiec, Poland; bober@sum.edu.pl; 3 Department of Pharmacodynamics, Medical University of Bialystok, Mickiewicza 2c, 15-222 Bialystok, Poland; justyna.hermanowicz@umb.edu.pl; 4 Department of Clinical Pharmacy, Medical University of Bialystok, Mickiewicza 2c, 15-222 Bialystok, Poland; 5 Department of Organic Chemistry, Faculty of Pharmaceutical Sciences in Sosnowiec, Medical University of Silesia in Katowice, 4 Jagiellońska, 41-200 Sosnowiec, Poland; ebebenek@sum.edu.pl (E.B.); echrobak@sum.edu.pl (E.C.); 6 Department of Medicinal Chemistry, Medical University of Bialystok, Kilinskiego 1, 15-089 Bialystok, Poland; arkadiusz.surazynski@umb.edu.pl

**Keywords:** triterpenoids, lipophilicity, indole, RP-TLC, zebrafish

## Abstract

There are scientific studies indicating that the attachment of an indole moiety to the triterpene scaffold can lead to increased anticancer potential. Lipophilicity is one of the factors that may influence biological properties and is therefore an important parameter to determine for newly obtained compounds as drug candidates. In the present study, previously synthesized 3 and/or 28-indole-betulin derivatives were evaluated for lipophilicity by reversed-phase thin-layer chromatography. The experimental values of lipophilicity (logP_TLC_) were then subjected to correlation analysis with theoretical values of logP, as well as for selected physicochemical and pharmacokinetic parameters and anticancer activity. A toxicity test using zebrafish embryos and larvae was also conducted. High correlation was observed between the experimental and theoretical values of lipophilicity. We presented correlation equations and statistical parameters describing the relationships between logP_TLC_ and several physicochemical and ADME parameters. We also revealed the lack of correlation between the experimental values of lipophilicity and anticancer activity. Moreover, experiments on zebrafish have confirmed no toxicity of the tested compounds, which was consistent with the results of the in silico toxicity analysis. The results demonstrated, using the example of indole derivatives of betulin, the utility of lipophilicity values in the context of predicting the biological activity of new compounds.

## 1. Introduction

Natural and synthetic heterocyclic compounds constitute a promising group of substances with anticancer activity. The presence of heteroatoms (nitrogen, sulfur and oxygen) in the structure means that heterocyclic compounds can act as acceptors and donors of hydrogen bonds. Thanks to this, these compounds have the ability to bind to many receptors through intermolecular hydrogen bonds, which leads to an appropriate pharmacological effect. The current trend in the search for new, more effective and selective chemotherapeutics is the combination of pharmacophore systems with different mechanisms of action. In the synthesis of such conjugates, nitrogen heterocyclic systems such as pyrazole, piperazine, quinolines, triazole and indole are often used. It has been shown that molecules containing the indole moiety can exert anticancer effects through various molecular targets, such as kinases, tubulin, aromatase or DNA topoisomerase [1,2,3,4,5,6,7,8,9].

The indole derivatives of triterpenoids have shown significant anticancer activity against hepatocarcinoma (SMMC-7721, HepG2), melanoma (A5375), leukemia (U937, K562, Jurkat, SR), lung cancer (NCI-H460) and glioma (U251, C6) cell lines. Attachment of the indole moiety to the triterpene scaffold may lead to increased anticancer activity in relation to individual components and reduced drug resistance [9,10,11,12,13,14].

The search for new drugs with the desired biological properties is the subject of the work of many teams of scientists. They pay special attention to lipophilicity, as it is a property that plays a significant role when designing new drugs [15]. Lipophilicity, generally speaking, determines the ability of a compound to dissolve in oil, fats, lipids and other nonpolar solvents. Lipophilicity is expressed as a logP value. Studies on this property are very often based on calculations of the partition coefficient between n-octanol and water [16]. Computer methods are also used for this purpose, sometimes created by leading pharmaceutical companies. Lipophilicity is closely related to ADME properties: solubility, permeability, clearance, metabolism or bioavailability, and distribution. Lipophilicity is also one of the factors that may influence biological properties [17], particularly the solubility, reactivity or even degradation of drugs. It also determines transport through biological membranes. In the laboratory, lipophilicity can be determined classically using the shake-flask method, as well as solid-phase microextraction (SPME) or potentiometric titration. One of the indirect methods using chromatography, both RP-TLC (reversed-phase thin-layer chromatography) and HPLC (high-performance liquid chromatography), has many practical applications. LogP values are often predicted on the basis of various computational models [18]. These calculations can be based on the structure of compounds, using a whole range of topological indices, E-state descriptors or even neural networks.

The zebrafish is an important biomedical model that provides an opportunity to understand the pathogenesis of many human diseases, such as osteoporosis, inflammation, autism spectrum disorders, obesity, Type 2 diabetes, heart failure and cancers. The advantages of this model include the ability to obtain a large number of embryos in a single clutch and the optical transparency of the developing embryo, which illustrates the course of changes induced by various factors in a living organism. Zebrafish embryos are often used in toxicity studies because this species is as sensitive to teratogenic compounds as mammals. The zebrafish model also allows for the rapid assessment of in vivo toxicity mechanisms induced by newly discovered chemical compounds considered as drug candidates [19,20,21,22,23].

In this study, previously synthesized indole derivatives of botulin, shown in Table 1, were assessed for lipophilicity by the reversed-phase thin-layer chromatography method (RP-TLC).

Additionally, the experimental values of lipophilicity (logP_TLC_) were subjected to correlation analysis for theoretical values of logP, selected physicochemical and ADME parameters and anticancer activity. Indole derivatives that were the most active against MCF-7 cells were subjected to a toxicity test using zebrafish (*Danio rerio*) embryos and larvae.

## 2. Results and Discussion

### 2.1. Lipophilicity—Experimental and Theoretical Studies

Lipophilicity is a physicochemical property of a compound describing its behavior in a two-phase system consisting of a nonpolar organic phase and a polar phase, most often aqueous. This parameter is one of the key features of compounds required to assess the processes of absorption, distribution and transport in biological systems, next to solubility, stability and acid–base characteristics. Moreover, lipophilicity is a factor determining the affinity of the compound to the target proteins, which is responsible for the final effect of biological action [24,25,26].

The studies we conducted involved the use of reversed-phase thin-layer chromatography (RP-TLC) to determine the experimental logP_TLC_ values of previously synthesized 3 and/or 28-indole-betulin derivatives (Table 1) [27,28].

The initial stage of the studies aimed at obtaining the logP_TLC_ values of the tested compounds concerned the determination of the standard curve. For this purpose, the lipophilicity values were determined experimentally (R_M0_), and values from the literature (logP_lit_) [29,30] were used for the following standard substances: acetanilide, prednisone, 4-bromoacetophenone, benzophenone, anthracene, dibenzyl and DDT (dichlorodiphenyltrichloroethane). Determination of the R_M0_ values for reference compounds was performed under the same chromatographic conditions as for the indole derivatives EB355A, EB365, EB366 and EB367. The standard curve of the relationship between logP_lit_ and R_M0_ is described by the following equation:

LogP_TLC_ = 1.1332 R_M0_ + 0.6683 (*r* = 0.992) (1)

Table 2 presents the literature-based (logP_lit_) and experimental values of lipophilicity (R_M0_ and logP_TLC_) for the reference substances.

Using the equations R_M_ = R_M0_ + bC and logP_TLC_ = 1.1332R_M0_ + 0.6683 and the R_M_ values determined during the experiment for indole derivatives of betulin, logP_TLC_ values were calculated. These values are presented in Table 3.

The experimentally determined logP_TLC_ values for the indole derivatives of betulin ranged from 7.52 to 8.28. The highest lipophilicity among the compounds tested was characterized by the diester derivative EB365, which had an acetyl substituent at the C-3 position, and the lowest was characterized by the monoester derivative EB355A containing a free hydroxyl group at the C-3 carbon atom. Both of these derivatives have an indolyl moiety at the C-28 position. The indole derivatives EB355A and EB367, containing a free hydroxyl group at positions C-3 and C-28, respectively, are characterized by reduced lipophilicity compared with the disubstituted derivatives EB365 and EB366. In the conducted study, betulin was used as a reference compound (logP_TLC_ = 6.11).

The results obtained by us confirmed previous studies on the lipophilicity parameters of betulin’s mono- and diester derivatives. The lower values of the lipophilicity parameter of betulin’s monoester derivatives resulted from the presence of a hydrophilic hydroxyl group attached to the hydrophobic triterpene scaffold [31,32].

Except for the lipophilicity values obtained from the thin-layer chromatography experiment, the lipophilicity values obtained using computer programs or Internet databases (iLOGP, XLOPG2, XLOPG3, WLOGP, MLOGP, SILICOS-IT, milogP, ACD/logP, KOWWIN and ALOGPs) were considered as well. These values of the theoretical lipophilicity for the analyzed triterpenoids are presented in the Table 4.

**Figure 1:**
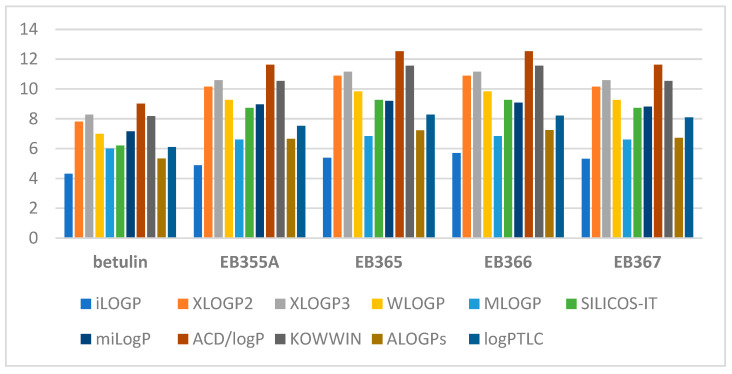
A comparison of the lipophilicity values obtained for the tested triterpenoids.

The theoretical values of the logP parameter of the indole derivatives of betulin ranged from 4.89 to 12.52. The graph below (Figure 1) shows a comparison of the experimentally determined values of lipophilicity with the theoretical values obtained using various commercially available computer programs.

By analyzing the above graph, it can be concluded that the lowest lipophilicity values are characteristic of betulin (logP = 4.31–8.28). The modification of the betulin molecule caused all its derivatives to have higher lipophilicity values. iLOGP predicted the lowest values for all compounds, and ACD/logP had the highest values. The experimental logP_TLC_ value of betulin’s indole derivatives was closest to the theoretical value obtained using ALOGP.

### 2.2. Correlation Analysis

Table 5 presents the results of the correlation analysis between all values considered in the studies. These include the lipophilicity value determined experimentally (logP_TLC_) and 10 lipophilicity values determined theoretically using generally available online calculators. The correlations between logP_TLC_ and other values were specific for the analysis. High correlation values (all above 0.9) allowed us to set the correlation equations to calculate logP_TLC_ based only on the theoretical value.

A correlation analysis was also performed between the logP_TLC_ parameters and the values of the physicochemical (M, molar weight; TPSA, topological polar surface; nROTB, rotational bonds; nHBD, hydrogen bond donors; nHBA, hydrogen bond acceptors; MR, molar refractivity) and ADME properties (logPapp, Caco-2 permeability; logKp, skin permeability) (Table 6).

And in this case (Table 6), all correlation coefficients, except nHBD, were also high. In the case of this parameter, it was impossible to find a correlation equation that would describe the linear relationship between logP_TLC_ and nHBD. In this case, the correlation coefficient was 0.608 and the significance level was *p* = 0.28. As can be seen in Table 6, the number of donors for betulin and the EB355A and EB367 derivatives was the same, while the compounds differed in lipophilicity (logP_TLC_ value). This situation resulted from introducing a lipophilic indole system and replacing one of the donors (OH in Position C-3 or C-28) with an NH group of similar acidity. In the case of excluding betulin from the calculations, a higher correlation coefficient was obtained from the dependence of logP_TLC_ on nHBD. Another parameter of hydrogen bonds assumed in the calculations was the number of hydrogen bond acceptors (nHBA), which were different for betulin and its indole derivatives, for which a high correlation was obtained. Table 7 presents all statistically significant correlation equations and the parameters describing them. Due to the high values of the correlation coefficients, all equations presented here could be of use for determining the lipophilicity of the tested compounds without the need to conduct an experiment.

### 2.3. Cluster Analysis (CA)

**Figure 2:**
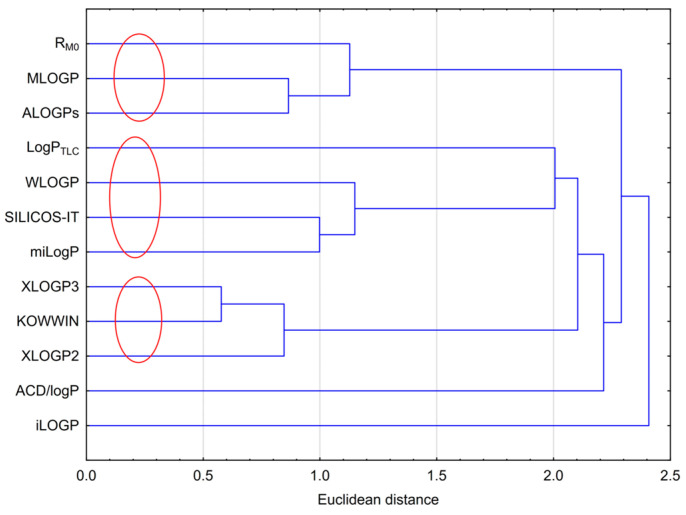
Analysis of similarities between the experimental and theoretical logP values.

Cluster analysis for the results obtained, including the experimental lipophilicity (R_M0_ and logP_TLC_) and those calculated using computer programs, was performed. The analysis is presented in Figure 2. Figure 2 shows three visible clusters that grouped the results obtained. The first one included R_M0_, MLOGP and ALOGPs; the second included logP_TLC_, WLOGP, SILICOS-IT and milogP; and the third one consisted of XLOGP2, XLOGP3 and KOWWIN. The grouping arose from the similarity in the individual values of lipophilicity. The values from WLOGP, SILICOS-IT and milogP were the most similar to the experimental logP_TLC_ value. ACD/logP and iLOGP were the ones that deviated the most from the rest, which was obviously due to their values. The ACD/logP values were the highest, and iLOGP was the lowest among the others.

**Figure 3:**
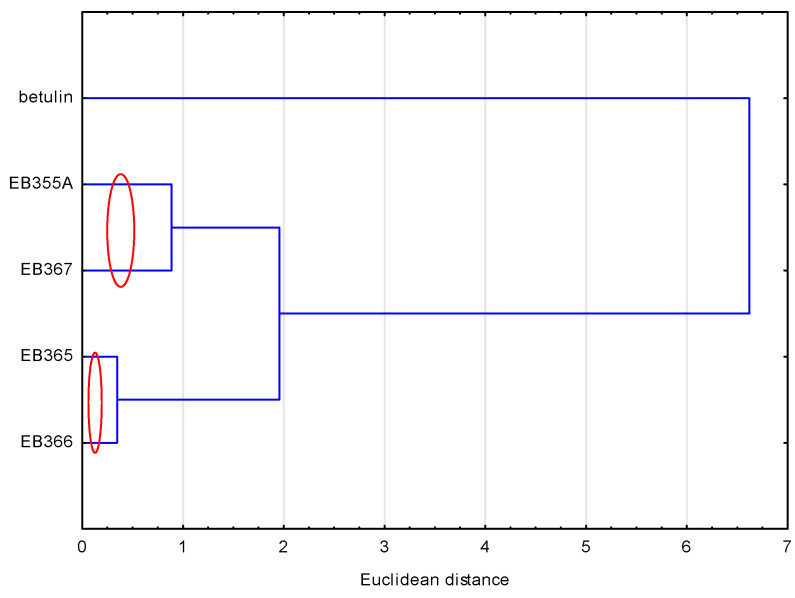
Analysis of similarities of the analyzed compounds, based on their lipophilicity values.

To compare the compounds analyzed, another similarity analysis was performed. This analysis showed (Figure 3) that the compounds formed two pairs, i.e., EB355A and EB367, and EB365 and EB366, which resulted directly from their structure. The first two had a free OH group in the C-3 and C-28 positions, and the remaining two had an acetyl group in analogous positions. Betulin, because its structure differed the most from other newly synthesized compounds, did not belong to any group.

**Figure 4:**
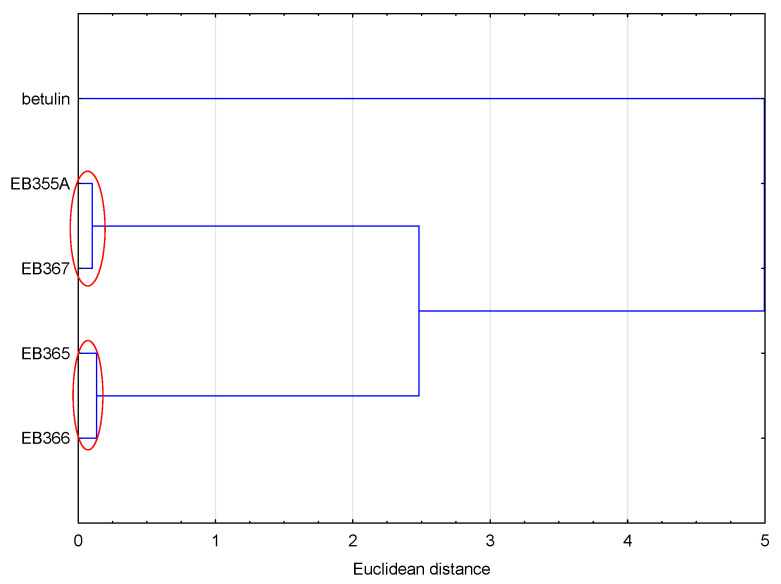
Analysis of similarities of the analyzed compounds, based on the values of their physicochemical and ADME properties.

Similar conclusions as in the case of lipophilicity were obtained by analyzing all the values of the physicochemical properties and ADME values (Figure 4). The compounds were grouped in the same way as in the case of the analysis based on the lipophilicity values. Because the values of the analyzed data were calculated according to the SMILE code, which resulted directly from the structure of the compound, compounds with similar structures formed one cluster.

### 2.4. Principal Component Analysis (PCA)

**Figure 5:**
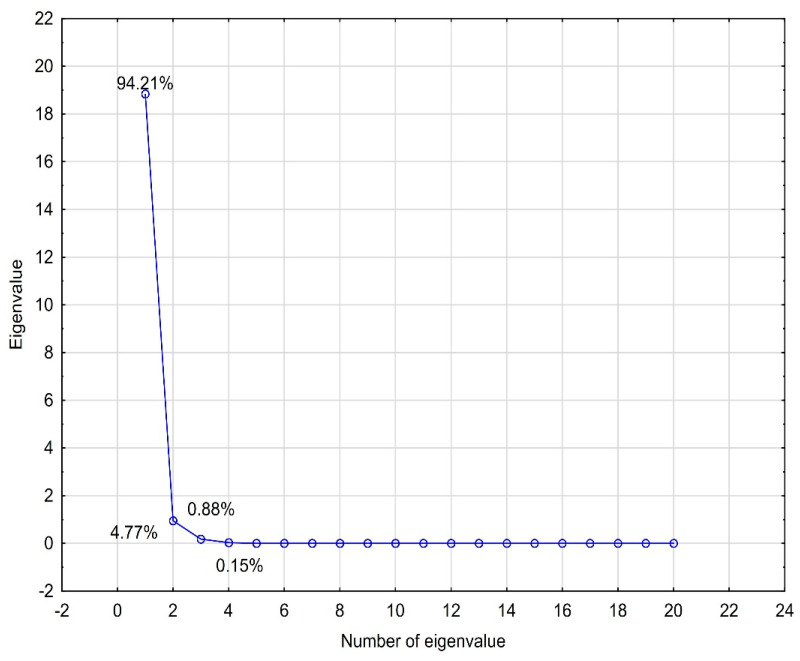
Scree plot obtained for the lipophilicity, ADME and physicochemical values of the compounds investigated.

**Figure 6:**
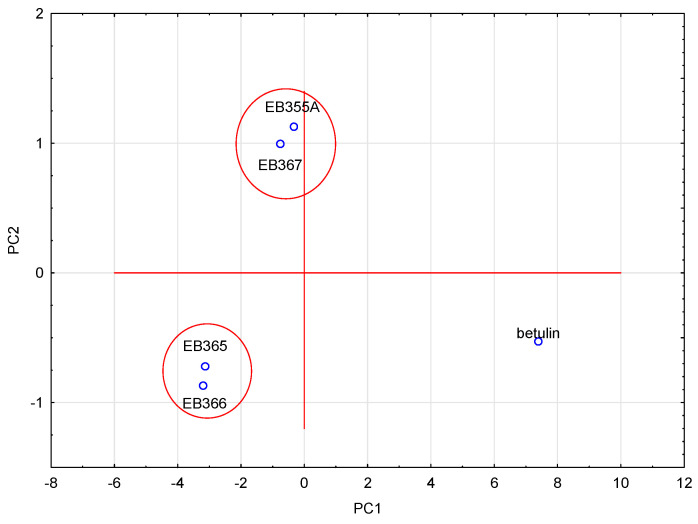
Projection of cases on the plane of factors for the PCA of the logP, physicochemical and ADME values of the analyzed compounds.

PCA analysis was performed for the logP values (experimental and theoretically calculated), physicochemical properties and ADME properties. The analyzed data were standardized. Four eigenvalues, which described 100% of the variability of the system, were obtained. To select the number of eigenvalues, a scree plot was used (Figure 5). The first eigenvalue had a very high percentage of the total variance (94.21%). The remaining eigenvalues complemented it. When we analyzed the contributions of individual data values to the main components, it was visible that the first component contained the highest shares of almost all data. Only the nHBD value made the highest contribution to the second eigenvalue. A graph of the cases projected onto the factor plane was prepared using the obtained eigenvalues (Figure 6). The analyzed compounds were grouped in pairs, i.e., EB355A and EB367, and EB365 and EB366. The same pairs of compounds formed clusters when analyzing the lipophilicity values using CA. The compounds that formed pairs on the plot had very similar structures, and betulin, which differed from the others, was located in a completely different part of the chart. The relationships discussed here are presented in the figures below.

### 2.5. Correlation with IC_50_ Values

The cytotoxic activity of indole-functionalized betulin derivatives was tested in vitro against amelanotic melanoma (A375, C32), triple-negative breast cancer (MDA-MB-231), estrogen receptor-positive breast cancer (MCF-7), lung cancer (A549) and colon adenocarcinoma (DLD-1, HT-29) cells [27,28].

A correlation analysis of all the values of lipophilicity, ADME, physicochemical properties and IC_50_ values, describing the cytotoxic activity of indole derivatives of betulin, was also performed. Due to the fact that only three of the tested compounds (EB355A, EB366 and EB367) had cytotoxic activity, such a correlation was performed only for them. Another limitation was the cell lines against which the abovementioned compounds showed cytotoxicity; therefore, the correlation applied only to the MCF-7 cell line (estrogen-receptor-positive breast cancer). The IC_50_ values of the EB355A, EB366 and EB367 derivatives against MCF-7 cells were 67, 156 and 35 µM, respectively. The correlation analysis between the IC_50_ values for MCF-7 and the other analyzed values for the compounds EB355A, EB366 and EB367 is presented in Table 8.

As Table 8 shows, the correlation coefficients had low values. The highest value was for the relationship between the IC_50_ for MCF-7 and milogP. Most of the analyzed data were determined on the basis of the structure. Hence, we could conclude that the structure of the compound is not so significant for the IC_50_ value. However, the lack of a correlation with the experimental values of lipophilicity (R_M0_ and logP_TLC_) indicated no dependence between these two properties, i.e., logP does not depend on IC_50_ nor does IC_50_ depend on logP.

### 2.6. In Silico Analysis of Toxicity 3- and 28-Indolobetulin Derivatives

The development of new drugs is a costly and lengthy process, often burdened with failure due to the occurrence of toxicity of the bioactive substance. The toxic effects are most often observed at late stages of the development of the drug. Therefore, there is a need for a rapid assessment of the toxicity of a chemical compound in the early stages of research. For this purpose, various in silico predictions are used, which allow us, among other matters, to determine toxicity at the organ level (hepatotoxicity, nephrotoxicity, neurotoxicity, cardiotoxicity and skin toxicity) [33,34]. Predictions of toxicity for indole derivatives of betulin were performed using the web tool admetSAR 3.0 [35,36] (Table 9).

On the basis of the data obtained, a compound is classified as nontoxic when the label is 0 or as toxic when the label is 1. All compounds were characterized by the absence of hepatotoxic, nephrotoxic, neurotoxic and skin-sensitizing effects. In silico prediction showed that only the 28-indolosubstituted derivative EB355A may exhibit cardiotoxic activity.

### 2.7. The Effects of EB355A or EB367A on the Development of Zebrafish Embryos/Larvae 

#### 2.7.1. Embryos of 0–2 hpf

Zebrafish embryos and larvae are good models for assessing the embryotoxic and teratogenic toxicity of biologically active substances. This model allows us to determine whether the tested compound is a teratogen, i.e., a substance that causes the development of a congenital anomaly or increases the occurrence of a specific hereditary deficiency. Analysis of the teratogenic effects of tested compounds is easy to perform due to the transparency of zebrafish embryos, including tail and notochord malformations, pericardial and yolk edema, scoliosis and growth retardation [37].

**Figure 7:**
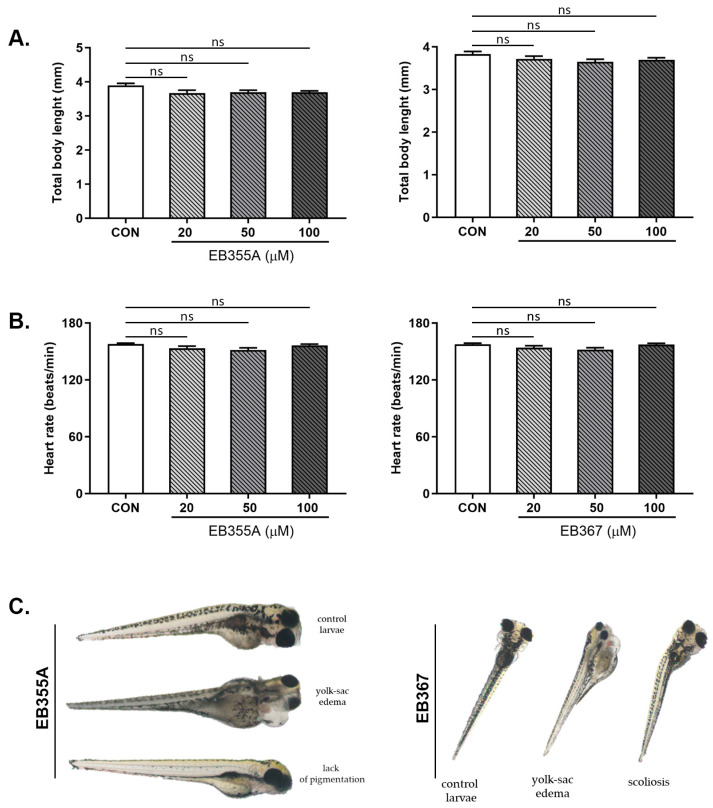
Total body length (**A**), heart rate (**B**) and sublethal alternations (**C**) of zebrafish embryos at 0–2 hpf exposed to EB355A, EB367 or the control (CON) for 96 h. Data are shown as the mean ± SD; n = 60 for each concentration. Abbreviations: hpf, hours post-fertilization; ns, not significant.

The effects of the compounds EB355A or EB367 on survival and early embryonic development were studied at 4, 8, 12, 24, 48, 72 and 96 h after exposure. No significant differences were noted between untreated embryos and embryos incubated with tested compounds. Treatment of the embryos with EB355A or EB367 did not affect the heart rate of the striped danio, reflected as the HR (heart rate), compared with the control embryos (Figure 7).

#### 2.7.2. Larvae of 72 hpf

**Figure 8:**
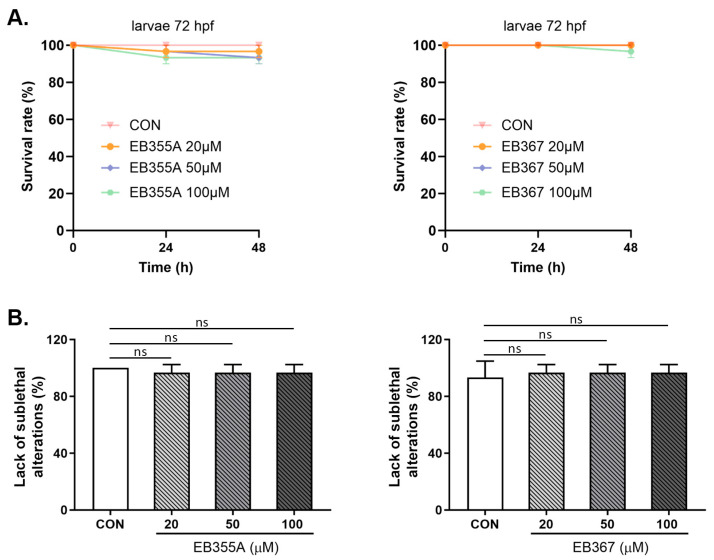
Survival rate (**A**) and lack of sublethal alternations (**B**) for larvae at 72 hpf exposed to EB355A, EB367A, or the control (CON) for 24 h and 48 h. Data are shown as the mean ± SEM; n = 60 for each concentration. Abbreviations: hpf, hours post-fertilization; ns, not significant.

A similar effect to the embryo study was obtained when both EB355A and EB367 were tested in 72 hpf larvae. Both compounds, at all concentrations used, did not cause changes in the tested larvae’s parameters with respect to the control larvae. No statistically significant growth defects or developmental abnormalities were observed under exposure to the tested compounds during the assessment to the endpoint (Figure 8).

## 3. Materials and Methods

### 3.1. Chemicals

The reagents and solvents applied in the synthesis of the tested compounds (EB355A, EB365, EB366 and EB367) and the chromatography materials were purchased from Merck (Darmstadt, Germany). Indole derivatives were obtained under the conditions of the Steglich method using betulin, 3-acetylbetulin, 28-acetylbetulin and 28-tetrahydropyranyl ether of betulin as substrates, which were reacted with 3-indoleacetic acid in the presence of DCC (*N*,*N*′-dicyclohexylcarbodiimide) and DMAP (4-dimethylaminopyridine). The 3- and 28-indolobetulin derivatives were obtained with yields ranging from 48% to 58%. The synthesis and chemical structure of the studied indole betulin derivatives have been described previously in the literature [27,28].

### 3.2. Thin-Layer Chromatography

An experiment for determining the lipophilicity with use of RP-TLC was carried out on plates precoated with silica gel RP-18F_254_ (#1.05559, Merck, Darmstadt, Germany). The horizontal chamber in which the plates were developed was previously saturated with vapor of the mobile phase. The mobile phase was mixture of acetone (Merck, Darmstadt, Germany) and a 0.2 M Tris buffer (Merck, Darmstadt, Germany) characterized by pH = 7.4. The volume ratio was 90:10, 85:15, 80:20, 75:25, 70:30, 65:35 and 60:40 for the reference compounds, and 90:10, 85:15, 80:20, 75:25, 70:30 and 65:35 for the betulin derivatives. The average values of the retardation factor (R_F_) were calculated and converted to R_M_ values according to the equation:

(2) $$R_{M}=\log\left(\frac{1}{R_{F}}-1\right)$$

The R_M_ values were linearly dependent on the acetone content in the mobile phase. The R_M0_ was also calculated according to the equation

R_M_ = R_M0_ + bC (3)

by extrapolating the concentration of acetone to zero. In the Equation (3), C is the concentration of acetone (% *v*/*v*) in the mobile phase whilst b is the regression term.

### 3.3. In Silico Studies

Many values of theoretical lipophilicity were used for the analysis. These were as follows.

- iLOGP, XLOGP3, WLOGP, MLOGP and SILICOS-IT were obtained on the basis of the SMILE code for compounds investigated using the web tool operated by the Molecular Modelling Group of the University of Lausanne and the SIB Swiss Institute of Bioinformatics [38,39];
- XLOPG2, ALOGPs data were obtained with the use of the Virtual Computational Chemistry Laboratory [40,41];
- MilogP data were obtained by using Molinspiration Free Services, based on the structural formula [42];
- KOWWIN data were obtained by using the freely available computer program EPIWEB 4.1;
- ACD/logP data were based on the structural formula using the program ChemSketch ACD/Chem Sketch(Freeware) 2021.2.1.

Other data (i.e., selected physicochemical and ADME properties of betulin and its derivatives) were obtained in the same way as some values of lipophilicity with the use of web tools [38,39]. They were as follows: molar weight (M), topological surface area (TPSA), the number of rotatable bonds (nROTB), the number of the hydrogen bond donors (nHBD), the number of hydrogen bond acceptors (nHBA), molar refractivity (MR), Caco-2 permeability (logPapp) and skin permeability (logKp).

### 3.4. Chemometric Data Analysis

In order to analyze and evaluate the results obtained for the lipophilicity values, the physicochemical and ADME properties of the tested compounds, a correlation analysis, a similarity analysis (CA) and a principal component analysis (PCA) were performed. On the basis of the correlation analysis, correlation equations and their statistical parameters were determined. Similarity analysis allowed us to group the received data in terms of their similarity. During the analysis, the calculations were based on Euclidean distances and single linkage distances [43]. Principal component analysis, on the other hand, allowed the reduction in the number of variables to the smallest possible number necessary to describe the system [44]. The number of main components was determined on the basis of scree plots. All data used for the PCA were standardized. The analyses were performed using Statistica 13.3 software.

### 3.5. Zebrafish Husbandry

The zebrafish embryos were maintained at a temperature of 28.0 ± 1.0 °C and a light/dark cycle, which was in accordance with the guidelines of the Department of Research Animals of the esteemed RSPCA (Royal Society for the Prevention of Cruelty to Animals). According to EU Directive 2010/63/EU, the earliest life stages of the striped danios (embryo and eleuteropod embryo cultures) are considered to be equivalent to in vitro cell cultures; therefore, they are not subject to the regulatory framework for animal experimentation. The embryos and larvae of striped danios were used before 120 hpf (hours after fertilization); therefore, ethical approval was not required. The embryos of striped danios were obtained by mating adult striped danios.

#### 3.5.1. Zebrafish Toxicity Assay

The fish embryo toxicity (FET) test was performed with modifications. Fertilized wild-type (WT) zebrafish embryos (0–2 hpf) or 72 hpf larvae were transferred to 6-well plates with a standard E3 medium and a series of EB355A or EB367 test compounds at concentrations of 20, 50 and 100 μM. Dimethylsulfoxide (DMSO, Sigma-Aldrich) was used as a solvent. The final concentration of DMSO in the solutions did not reach toxic concentrations above 1%. Control embryos were incubated in an embryo medium in the presence of 1% DMSO. Control and treated embryos were examined after 4, 8, 12, 24, 48, 72 and 96 h of treatment under a stereomicroscope with a camera. Experiments were conducted in triplicate, and 20 embryos were used in each group. Up to four apical observations were recorded every 24 h as indicators of mortality: coagulation of fertilized eggs, lack of somite formation, lack of detachment of the tail bud from the yolk sac and lack of a heartbeat. Pigmentation at 48 h was also observed. Additional developmental changes (heart rate, total body length) and embryonic malformations, such as pericardial edema, yolk sac edema, tail curvature, somite formation and scoliosis, were checked after 96 h.

For the toxicity test with 72 hpf larvae, three replicates were performed. For each replicate, 20 objects were used for each concentration and 20 larvae were used as controls (1% DMSO). The larvae were monitored at 24 and 48 h after treatment with different concentrations of EB355A or EB367 compounds. The survival rate and morphological deformities were examined and documented using a stereomicroscope equipped with a camera. After completing the observations, all remaining embryos/larvae were euthanized using a buffered tricaine methanesulphonate solution as per the OECD test guideline 236 (Organization for Economic Co-operation and Development 2013).

#### 3.5.2. Statistical Analysis

Shapiro–Wilk’s W test of normality was used for the analysis of data distribution. The normally distributed data were analyzed using a one-way analysis of variance (ANOVA) and shown as the mean ± SEM. The statistical analysis was conducted using GraphPad Prism 9.4 software (version 9.4.1). The differences were deemed statistically significant at *p* < 0.05.

## 4. Conclusions

In the present study, we revealed high correlations between the experimental and theoretical values of lipophilicity obtained for indole derivatives of betulin. We also presented the relationships between lipophilicity and the physicochemical and ADME parameters, as well as the lack of a correlation between the experimental values of the lipophilicity of the derivatives and their anticancer activity against MCF-7 cells. Moreover, our experiments on zebrafish confirmed that the tested compounds are not toxic, which was consistent with the results of in silico toxicity analysis. In summary, the study demonstrated, using the example of indole derivatives of betulin, the utility of lipophilicity values in the context of predicting the biological activity of new compounds.

## Figures and Tables

**Table 1:** The chemical structures of indole-functionalized derivatives of betulin.

Compound	Chemical Structure
EB355A	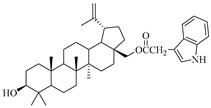
EB365	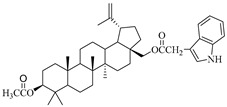
EB366	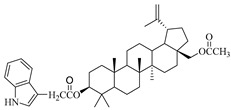
EB367	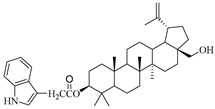

**Table 2:** The experimental (R_M0_ and logP_TLC_) and literature-based (logP_lit_) lipophilicity values for reference compounds (mobile phase: acetone–Tris buffer).

Reference Compound	R_M0_	LogP_lit_	b	r	LogP_TLC_
Acetanilide	0.57	1.21	−0.01	0.982	1.31
Prednisone	0.72	1.62	−0.01	0.989	1.48
4-Bromoacetophenone	1.82	2.43	−0.02	0.985	2.73
Benzophenone	2.23	3.18	−0.03	0.989	3.19
Anthracene	3.03	4.45	−0.04	0.993	4.10
Dibenzyl	3.51	4.79	−0.04	0.994	4.64
DDT	5.20	6.38	−0.06	0.997	6.56

b is the slope and r is the correlation coefficient for the linear relationship R_M_ = R_M0_ + bC.

**Table 3:** The experimental values of lipophilicity for the tested derivatives in the mobile phase (acetone–Tris buffer).

Compound	R_M0_	b	r	LogP_TLC_
Betulin	4.81	−0.05	0.989	6.11
EB355A	6.05	−0.07	0.989	7.52
EB365	6.72	−0.07	0.993	8.28
EB366	6.66	−0.07	0.995	8.21
EB367	6.55	−0.07	0.985	8.09

b is the slope and r is the correlation coefficient for the linear relationship R_M_ = R_M0_ + bC.

**Table 4:** The theoretical values of lipophilicity for betulin and its indole derivatives.

Compound	iLOGP	XLOPG2	XLOGP3	WLOGP	MLOGP	SILICOS-IT	MilogP	ACD/logP	KOWWIN	ALOGPs
Betulin	4.31	7.82	8.28	7.00	6.00	6.21	7.16	9.01	8.18	5.34
EB355A	4.89	10.15	10.58	9.27	6.61	8.74	8.96	11.62	10.54	6.65
EB365	5.39	10.89	11.15	9.84	6.85	9.27	9.20	12.52	11.55	7.22
EB366	5.70	10.89	11.15	9.84	6.85	9.27	9.08	12.52	11.55	7.25
EB367	5.32	10.15	10.58	9.27	6.61	8.74	8.81	11.62	10.54	6.73

**Table 5:** Values of the coefficients for the correlation analysis between the experimental and theoretical values of lipophilicity for compounds investigated (mobile phase: acetone–Tris buffer).

	iLOGP	XLOGP2	XLOGP3	WLOGP	MLOGP	SILCOS-IT	MilogP	ACD/logP	KOWWIN	ALOGPs	LogP_TLC_
**iLOGP**	1.000	0.926	0.917	0.917	0.933	0.910	0.865	0.929	0.935	0.946	0.953
**XLOGP2**		1.000	0.997	0.998	0.998	0.996	0.986	0.999	0.997	0.997	0.974
**XLOGP3**			1.000	1.000	0.994	0.999	0.993	0.997	0.991	0.992	0.974
**WLOGP**				1.000	0.994	0.999	0.992	0.997	0.991	0.992	0.975
**MLOGP**					1.000	0.989	0.977	0.999	0.999	0.999	0.970
**SILCOS-IT**						1.000	0.995	0.994	0.986	0.988	0.974
**MilogP**							1.000	0.983	0.972	0.972	0.954
**ACD/logP**								1.000	0.998	0.998	0.972
**KOWWIN**									1.000	0.999	0.967
**ALOGPs**										1.000	0.978
**LogP_TLC_**											1.000

**Table 6:** Selected physicochemical and ADME properties of the tested triterpenoids.

Compound	M (g/mol)	TPSA (Å^2^)	nROTB	nHBD	nHBA	MR ^a^	LogPapp ^b^	LogKp ^c^
Betulin	442.72	40.46	2	2	2	136.30	1.375	−2.789
EB355A	599.89	62.32	6	2	3	182.38	0.630	−2.736
EB365	641.92	68.39	8	1	4	192.12	0.674	−2.732
EB366	641.92	68.39	8	1	4	192.12	0.708	−2.730
EB367	599.89	62.32	6	2	3	182.38	0.660	−2.735

^a^ Molar refractivity; ^b^ Caco-2 permeability (logPapp in 10^−6^ cm/s); ^c^ skin permeability.

**Table 7:** Correlation equations and statistical parameters describing the relationships between logP_TLC_ and other data obtained.

Parameter	Equation	r	s	F	P
iLOGP	LogP_TLC_ = 1.606 (±0.296) iLOGP − 0.584 (±1.523)	0.953	0.319	29	0.01
XLOGP2	LogP_TLC_ = 0.699 (±0.095) XLOGP2 + 0.662 (±0.951)	0.974	0.239	55	0.00
XLOGP3	LogP_TLC_ = 0.742 (±0.098)XLOGP3 − 0.042 (±1.025)	0.974	0.235	57	0.00
WLOGP	LogP_TLC_ = 0.751 (±0.100) WLOGP + 0.852 (±0.907)	0.975	0.234	57	0.00
MLOGP	LogP_TLC_ = 2.529 (±0.368) MLOGP − 9.011 (±2.425)	0.970	0.256	47	0.01
SILICOS-IT	LogP_TLC_ = 0.691 (±0.093) SILICOS-IT+1.802 (±0.795)	0.974	0.238	55	0.00
MilogP	LogP_TLC_ = 1.029 (±0.186) milogP − 1.253 (±1.618)	0.954	0.314	30	0.01
ACD/logP	LogP_TLC_ = 0.612 (±0.085) ACD/logP + 0.624 (±0.976)	0.972	0.244	52	0.01
KOWWIN	LogP_TLC_ = 0.637 (±0.097) KOWWIN + 0.970 (±1.019)	0.967	0.266	43	0.01
ALOGPs	LogP_TLC_ = 1.1415 (±0.147) ALOGPs + 0.065 (±0.982)	0.978	0.228	60	0.00
M	LogP_TLC_ = 0.011 (±0.001) M + 1.362 (±0.839)	0.975	0.234	57	0.00
TPSA	LogP_TLC_ = 0.077 (±0.010) TPSA + 3.016 (±0.623)	0.975	0.235	57	0.00
nROTB	LogP_TLC_ = 0.354 (±0.059) nROTB + 5.507 (±0.379)	0.961	0.291	36	0.01
nHBA	LogP_TLC_ = 0.978 (±0.270) nHBA + 4.513 (±0.889)	0.902	0.453	13	0.04
MR	LogP_TLC_ = 0.038 (±0.005) MR + 0.928 (±0.911)	0.974	0.238	55	0.00
LogPapp	LogP_TLC_ = −2.619 (±0.661) logPapp + 9.761 (±0.567)	0.916	0.419	16	0.03
LogKp	LogP_TLC_ = 34.913 (±5.572) logK_p_ + 103.458 (±15.294)	0.964	0.279	39	0.01

**Table 8:** Correlation analysis between the IC_50_ values for MCF-7 and the other values analyzed for the compounds EB355A, EB366 and EB367.

MCF-7 [IC_50_ µM]		MCF-7 [IC_50_ µM]	
R_M0_	0.340	KOWWIN	0.262
LogP_TLC_	0.342	ALOGPs	0.219
iLOGP	−0.123	M [g/mol]	0.262
XLOGP2	0.262	TPSA [Å^2^]	0.262
XLOGP3	0.262	nROTB	0.262
WLOGP	0.262	nHBD	−0.262
MLOGP	0.262	nHBA	0.262
SILCOS-IT	0.262	MR	0.262
milogP	0.710	LogPapp	−0.183
ACD/logP	0.262	LogKp	−0.068

**Table 9:** Selected toxicity parameters of the indole derivatives of betulin performed using the admetSAR 3.0 web tool.

Compound	Organ Toxicity				
	Hepatotoxicity	Nephrotoxicity	Neurotoxicity	Skin Sensitization	Cardiotoxicity (hERG 1 µM)
EB355A	0	0	0	0	1
EB365	0	0	0	0	0
EB366	0	0	0	0	0
EB367	0	0	0	0	0

## Data Availability

The raw data supporting the conclusions of this article will be made available by the authors on request.
